# Supplementary material for: Dietary Fatty Acid Composition Impacts the Fatty Acid Profiles of Different Regions of the Bovine Brain
Source: Animals (Basel). 2022 Oct 7;12(19):2696. doi: 10.3390/ani12192696 (PMC9559283; doi:10.3390/ani12192696)
Supplement: Supplementary file 1 [file animals-12-02696-s001.zip › animals-1917715-supplementary.pdf]

Table S1. Fatty acid profiles for bovine amygdala, hippocampus, and mid tectum.

Region/tract:	Amygdala			Hippocampus			Mid tectum		
Supplement:	Fish	Palm	SEM[a]	Fish	Palm	SEM	Fish	Palm	SEM
Fatty acid[b]	(mg fatty acid/ 100 mg of total fatty acids)								
C14:0	0.69	0.67	0.02	0.62	0.61	0.03	0.41	0.37	0.03
C16:0	20.89	20.77	0.21	19.42	19.35	0.27	16.65	16.74	0.43
C16:1 n-7	0.53	0.48	0.02*	0.46	0.42	0.02	0.42	0.38	0.02
C17:0	0.35	0.39	0.02	0.42	0.43	0.01	0.53	0.53	0.01
C18:0	21.76	21.37	0.200	20.64	20.52	0.16	19.30	19.14	0.34
C18:1 n-9	18.56	18.73	0.50	22.00	21.12	0.51	26.61	26.94	1.01
C18:1 n-7	3.95	4.05	0.04	4.47	4.39	0.07	5.39	5.91	0.16*
C18:2 n-6	0.40	0.36	0.02	0.43	0.36	0.03	0.34	0.29	0.03
C18:3 n-3	0.10	0.07	0.02	0.11	0.13	0.02	0.23	0.22	0.02
C20:0	0.16	0.14	0.014	0.20	0.20	0.02	0.33	0.35	0.02
C20:1 n-9	0.90	1.15	0.100	1.83	1.82	0.15	3.01	3.34	0.24
C20:2 n-6	1.01	0.92	0.07	1.78	1.37	0.12*	0.63	0.55	0.03
C20:3 n-6	0.54	0.58	0.07	1.05	0.92	0.08	1.00	0.81	0.04**
C20:4 n-6	7.94	8.89	0.22**	5.95	7.51	0.26**	5.26	5.36	0.41
C20:5 n-3	0.68	0.28	0.04**	0.57	0.29	0.03**	0.33	0.21	0.02**
C22:0	0.12	0.12	0.02	0.24	0.24	0.02	0.49	0.57	0.04
C22:1 n-9	0.88	0.97	0.08	1.87	1.61	0.11	0.61	0.62	0.04
C22:4 n-6	4.29	5.88	0.13**	3.12	4.86	0.14**	3.79	5.16	0.18**
C22:5 n-3	2.76	1.12	0.09**	2.03	0.90	0.07**	2.08	0.85	0.10**
C22:6 n-3	11.84	10.68	0.36*	9.75	9.26	0.30	8.06	8.22	0.39

[a]Standard error of the mean, n=10, five steers and five heifers.

[b]Number of carbon atoms : number of carbon-carbon double bonds; "n" indicates number of carbon atoms removed from the methyl end to the first carbon-carbon double bond.

[*] $P < 0.05$; [**] $P < 0.01$.

Table S2. Fatty acid profiles for bovine mid tegmentum, medulla, and pons.

Region/tract:	Mid tegmentum			Medulla			Pons		
Supplement:	Fish	Palm	SEM	Fish	Palm	SEM	Fish	Palm	SEM
Fatty acid	(mg fatty acid/ 100 mg of total fatty acids)								
C14:0	0.36	0.35	0.01	0.40	0.39	0.02	0.40	0.39	0.01
C16:0	14.53	14.59	0.20	12.72	12.07	0.41	13.46	13.62	0.20
C16:1 n-7	0.28	0.26	0.01	0.28	0.20	0.04	0.25	0.22	0.02
C17:0	0.59	0.56	0.01	0.67	0.64	0.03	0.65	0.59	0.02
C18:0	19.15	19.04	0.18	16.75	16.11	0.42	17.63	17.83	0.24
C18:1 n-9	31.81	30.63	0.31*	32.84	32.50	1.32	32.38	31.74	0.57
C18:1 n-7	4.86	4.91	0.07	4.15	4.10	0.11	4.44	4.51	0.08
C18:2 n-6	0.22	0.21	0.01	0.20	0.14	0.05	0.20	0.17	0.03
C18:3 n-3	0.30	0.29	0.01	0.47	0.47	0.03	0.36	0.34	0.02
C20:0	0.42	0.41	0.01	0.62	0.66	0.05	0.44	0.46	0.03
C20:1 n-9	5.08	5.26	0.25	8.48	9.20	0.61	6.56	6.84	0.24
C20:2 n-6	0.86	0.81	0.04	0.64	0.75	0.03*	0.91	0.99	0.04
C20:3 n-6	0.94	0.91	0.04	0.95	1.02	0.06	1.01	0.92	0.03
C20:4 n-6	3.88	4.54	0.17*	2.35	2.37	0.26	2.96	3.57	0.10**
C20:5 n-3	0.32	0.23	0.01**	0.32	0.30	0.03	0.29	0.27	0.01
C22:0	0.80	0.85	0.05	1.41	1.55	0.11	0.88	1.03	0.08
C22:1 n-9	0.95	1.03	0.05	0.49	0.63	0.05	0.91	1.12	0.07*
C22:4 n-6	3.60	5.02	0.11**	1.86	2.57	0.17**	2.72	3.97	0.14**
C22:5 n-3	1.85	0.80	0.07**	1.33	0.54	0.08**	1.70	0.66	0.06**
C22:6 n-3	5.29	5.05	0.26	3.57	2.33	0.73	4.10	3.72	0.11*

[a]Standard error of the mean, n=10, five steers and five heifers.

[b]Number of carbon atoms : number of carbon-carbon double bonds; "n" indicates number of carbon atoms removed from the methyl end to the first carbon-carbon double bond.

[*] $P < 0.05$; [**]$P < 0.01$.

Table S3. Fatty acid profiles for bovine parietal lobe, frontal lobe, and internal capsule.

Region/tract:	Parietal lobe			Frontal lobe			Internal capsule		
Supplement:	Fish	Palm	SEM	Fish	Palm	SEM	Fish	Palm	SEM
Fatty acid	(mg fatty acid/ 100 mg of total fatty acids)								
C14:0	0.57	0.55	0.03	0.71	0.61	0.04	0.33	0.32	0.01
C16:0	19.21	19.13	0.45	20.37	18.60	0.70	13.75	13.48	0.18
C16:1 n-7	0.48	0.45	0.02	0.59	0.49	0.03*	0.22	0.20	0.01
C17:0	0.46	0.46	0.02	0.43	0.40	0.02	0.62	0.62	0.01
C18:0	21.47	21.37	0.19	20.75	19.19	0.70	19.54	19.26	0.18
C18:1 n-9	22.46	22.03	0.83	19.47	18.76	0.77	33.05	32.57	0.40
C18:1 n-7	4.37	4.58	0.06*	4.11	3.94	0.14	4.49	4.55	0.09
C18:2 n-6	0.49	0.46	0.03	0.61	0.51	0.04	0.17	0.16	0.01
C18:3 n-3	0.16	0.16	0.02	0.10	0.03	0.02*	0.31	0.31	0.01
C20:0	0.21	0.20	0.01	0.10	0.26	0.12	0.37	0.37	0.01
C20:1 n-9	1.96	2.07	0.22	1.23	1.46	0.10	5.24	5.79	0.21
C20:2 n-6	0.88	0.70	0.05*	0.86	0.66	0.06*	0.87	0.84	0.04
C20:3 n-6	0.75	0.67	0.02*	0.77	0.62	0.05*	0.85	0.84	0.06
C20:4 n-6	5.61	6.41	0.25*	5.84	6.37	0.28	4.20	4.38	0.16
C20:5 n-3	0.38	0.17	0.03**	0.36	0.11	0.04**	0.29	0.19	0.03*
C22:0	0.31	0.30	0.03	0.11	0.14	0.03	0.80	0.91	0.05
C22:1 n-9	1.00	0.97	0.06	0.72	1.34	0.35	1.62	1.59	0.11
C22:4 n-6	2.78	3.89	0.09**	2.50	3.61	0.14**	4.02	4.89	0.12**
C22:5 n-3	1.79	0.83	0.07**	2.00	0.77	0.07**	1.28	0.52	0.05**
C22:6 n-3	12.09	11.70	0.52	13.28	11.37	0.57*	3.42	2.92	0.17*

[a]Standard error of the mean, n=10, five steers and five heifers.

[b]Number of carbon atoms : number of carbon-carbon double bonds; "n" indicates number of carbon atoms removed from the methyl end to the first carbon-carbon double bond.

* $P < 0.05$; $^{**}P < 0.01$.

Table S4. Fatty acid profiles for bovine caudate, putamen, and cerebellum.

Region/tract:	Caudate			Putamen			Cerebellum		
Supplement:	Fish	Palm	SEM	Fish	Palm	SEM	Fish	Palm	SEM
Fatty acid	(mg fatty acid/ 100 mg total fatty acids)								
C14:0	0.55	0.60	0.03	0.49	0.52	0.03	0.49	0.56	0.02*
C16:0	19.12	19.74	0.40	17.98	18.45	0.51	16.32	17.86	0.55
C16:1 n-7	0.58	0.56	0.03	0.50	0.49	0.03	0.34	0.35	0.02
C17:0	0.49	0.46	0.01	0.52	0.49	0.02	0.55	0.58	0.02
C18:0	22.29	22.09	0.27	21.64	21.54	0.25	20.28	21.21	0.42
C18:1 n-9	19.61	18.06	1.17	22.97	21.79	1.11	23.08	21.72	0.97
C18:1 n-7	4.79	4.73	0.10	4.69	4.70	0.07	4.33	4.55	0.11
C18:2 n-6	0.55	0.55	0.05	0.57	0.55	0.05	0.69	0.66	0.06
C18:3 n-3	0.11	0.11	0.02	0.16	0.13	0.02	0.22	0.11	0.04*
C20:0	0.20	0.15	0.02	0.23	0.37	0.14	0.26	0.21	0.04
C20:1 n-9	1.38	1.23	0.26	1.90	1.65	0.29	3.87	3.25	0.54
C20:2 n-6	0.56	0.48	0.04	0.57	0.59	0.07	0.50	0.44	0.02
C20:3 n-6	1.23	1.10	0.04*	1.23	1.06	0.07	1.68	1.60	0.09
C20:4 n-6	8.05	9.41	0.47*	6.92	7.79	0.30*	4.36	6.29	0.26**
C20:5 n-3	0.44	0.21	0.03**	0.36	0.17	0.03**	0.26	0.09	0.02**
C22:0	0.19	0.15	0.04	0.28	0.23	0.04	0.46	0.41	0.12
C22:1 n-9	0.46	0.47	0.09	0.58	0.69	0.08	0.17	0.15	0.05
C22:4 n-6	4.07	5.53	0.13**	3.78	4.98	0.12**	1.49	2.35	0.07**
C22:5 n-3	2.46	1.13	0.09**	2.36	1.12	0.09**	1.74	1.03	0.07**
C22:6 n-3	11.24	10.90	0.57	10.14	9.90	0.70	11.86	12.88	0.93

[a]Standard error of the mean, n=10, five steers and five heifers.

[b]Number of carbon atoms : number of carbon-carbon double bonds; "n" indicates number of carbon atoms removed from the methyl end to the first carbon-carbon double bond.

* P < 0.05; ***P* < 0.01.

Table S5. Fatty acid profiles for bovine visual cortex, sensory cortex, and motor cortex.

Region/tract:	Visual cortex			Sensory cortex			Motor cortex		
Supplement:	Fish	Palm	SEM	Fish	Palm	SEM	Fish	Palm	SEM
Fatty acid	(mg fatty acid/ 100 mg total fatty acids)								
C14:0	0.62	0.61	0.02	0.56	0.53	0.02	0.54	0.56	0.01
C16:0	20.26	20.19	0.37	19.55	18.95	0.26	19.47	19.73	0.27
C16:1 n-7	0.51	0.46	0.02*	0.50	0.43	0.02**	0.49	0.46	0.06
C17:0	0.44	0.43	0.02	0.46	0.46	0.01	0.47	0.44	0.01*
C18:0	22.14	21.93	0.19	21.79	21.40	0.21	21.86	21.71	0.23
C18:1 n-9	19.94	19.69	0.66	21.44	22.34	0.56	21.90	20.88	0.51
C18:1 n-7	4.18	4.15	0.08	4.51	4.49	0.05	4.46	4.48	0.07
C18:2 n-6	0.49	0.43	0.02	0.51	0.44	0.03	0.51	0.47	0.03
C18:3 n-3	0.10	0.12	0.18	0.14	0.15	0.01	0.14	0.11	0.01**
C20:0	0.17	0.17	0.01	0.20	0.20	0.01	0.21	0.19	0.01
C20:1 n-9	1.31	1.43	0.18	1.68	2.16	0.12*	1.82	1.76	0.13
C20:2 n-6	0.79	0.65	0.05	0.74	.074	0.04	0.75	0.66	0.05
C20:3 n-6	0.77	0.66	0.02**	0.81	0.69	0.03*	0.79	0.66	0.03**
C20:4 n-6	6.26	7.29	0.28*	5.85	6.30	0.24	5.63	6.68	0.19**
C20:5 n-3	0.43	0.19	0.04**	0.35	0.17	0.02**	0.34	0.17	0.02**
C22:0	0.18	0.21	0.03	0.25	0.32	0.02*	0.26	0.26	0.02
C22:1 n-9	0.85	0.89	0.07	0.86	1.07	0.08*	0.88	0.91	0.08
C22:4 n-6	2.92	4.30	0.11**	2.78	3.93	0.15**	2.62	3.96	0.08**
C22:5 n-3	2.09	0.94	0.10**	1.89	0.80	0.08**	1.78	0.83	0.05**
C22:6 n-3	13.51	12.49	0.45	12.79	11.18	0.40*	12.74	12.19	0.45

[a]Standard error of the mean, n=10, five steers and five heifers.

[b]Number of carbon atoms : number of carbon-carbon double bonds; "n" indicates number of carbon atoms removed from the methyl end to the first carbon-carbon double bond.

* P < 0.05; $^{**}P < 0.01$.

Table S6. Fatty acid profiles for bovine thalamus and hypothalamus

Region/tract:	Thalamus			Hypothalamus		
Supplement:	Fish	Palm	SEM	Fish	Palm	SEM
Fatty acid	(mg fatty acid/ 100 mg total fatty acids)					
C14:0	0.39	0.40	0.03	0.42	0.51	0.04
C16:0	17.77	16.97	0.49	18.22	19.06	0.4
C16:1 n-7	0.37	0.33	0.03	0.38	0.39	0.02
C17:0	0.53	0.54	0.02	0.47	0.45	0.02
C18:0	20.48	20.16	0.25	20.33	20.02	0.33
C18:1 n-9	25.61	26.19	1.13	24.73	22.76	0.91
C18:1 n-7	4.79	4.79	0.04	4.68	4.69	0.09
C18:2 n-6	0.39	0.35	0.03	0.35	0.38	0.04
C18:3 n-3	0.21	0.20	0.03	0.11	0.13	0.02
C20:0	0.28	0.29	0.02	0.25	0.24	0.02
C20:1 n-9	2.66	3.405	0.38	2.34	1.95	0.24
C20:2 n-6	0.69	0.61	0.04	1.03	1.02	0.15
C20:3 n-6	0.97	0.91	0.04	0.96	0.87	0.03
C20:4 n-6	5.35	6.03	0.40	5.84	7.16	0.40
C20:5 n-3	0.31	0.19	0.02**	0.50	0.24	0.05*
C22:0	0.41	0.52	0.06	0.32	0.28	0.04
C22:1 n-9	0.62	0.65	0.06	1.02	1.16	0.18
C22:4 n-6	3.85	5.18	0.17**	5.05	6.21	0.36
C22:5 n-3	2.15	0.92	0.07**	2.58	1.22	0.11**
C22:6 n-3	9.68	8.07	0.62	8.27	8.46	0.30

[a]Standard error of the mean, n=10, five steers and five heifers.

[b]Number of carbon atoms : number of carbon-carbon double bonds; “n” indicates number of carbon atoms removed from the methyl end to the first carbon-carbon double bond.

* P < 0.05; $^{**}P < 0.01$.
